# Comparison of *Mycobacterium tuberculosis *isocitrate dehydrogenases (ICD-1 and ICD-2) reveals differences in coenzyme affinity, oligomeric state, pH tolerance and phylogenetic affiliation

**DOI:** 10.1186/1471-2091-6-20

**Published:** 2005-09-29

**Authors:** Sharmistha Banerjee, Ashok Nandyala, RaviPrasad Podili, Vishwa Mohan Katoch, Seyed E Hasnain

**Affiliations:** 1 Centre for DNA Fingerprinting and Diagnostics, ECIL Road, Nacharam, Hyderabad, 500076, India; 2 Central JALMA Institute for Leprosy, Tajganj, Agra 282001, India; 3 Jawaharlal Nehru Centre for Advanced Scientific Research (JNCASR), Jakkur, Bangalore 560012, India

## Abstract

**Background:**

*M.tb icd*-1 and *M.tb icd*-2, have been identified in the *Mycobacterium tuberculosis *genome as probable isocitrate dehydrogenase (ICD) genes. Earlier we demonstrated that the two isoforms can elicit B cell response in TB patients and significantly differentiate TB infected population from healthy, BCG-vaccinated controls. Even though immunoassays suggest that these proteins are closely related in terms of antigenic determinants, we now show that *M.tb icd*-1 and *M.tb icd*-2 code for functional energy cycle enzymes and document the differences in their biochemical properties, oligomeric assembly and phylogenetic affiliation.

**Results:**

Functionally, both *M.tb *ICD-1 and ICD-2 proteins are dimers. Zn^+2 ^can act as a cofactor for ICD-1 apart from Mg^+2^, but not for ICD-2. ICD-1 has higher affinity for metal substrate complex (Km (isocitrate) with Mg^++^:10 μM ± 5) than ICD-2 (Km (isocitrate) with Mg^++^:20 μM ± 1). ICD-1 is active across a wider pH range than ICD-2, retaining 33–35% activity in an acidic pH upto 5.5. Difference in thermal behaviour is also observed with ICD-2 being active across wider temperature range (20°C to 40°C) than ICD-1 (optimum temperature 40°C). The isozymes are NADP^+ ^dependent with distinct phylogenetic affiliations; unlike *M.tb *ICD-2 that groups with bacterial ICDs, *M.tb *ICD-1 exhibits a closer lineage to eukaryotic NADP^+ ^dependent ICDs.

**Conclusion:**

The data provide experimental evidence to show that the two open reading frames, Rv3339c (ICD-1) and Rv0066c (ICD-2), annotated as probable ICDs are functional TCA cycle enzymes with identical enzymatic function but different physio-chemical and kinetic properties. The differences in biochemical and kinetic properties suggest the possibility of differential expression of the two ICDs during different stages of growth, despite having identical metabolic function.

## Background

The central metabolic pathways in bacteria, especially in *E.coli*, have been extensively studied to understand the physiology of the organisms under altered carbon sources [1]. One of the key regulatory enzymes in the universal tri-carboxylic acid energy cycle is the isocitrate dehydrodenase that allosterically regulates the conversion of oxidative decarboxylation of D-isocitrate to α-ketoglutarate and CO_2 _in presence of a cofactor [2]. This rate-limiting step is the first NADPH yielding reaction of the TCA cycle [2]. Isocitrate dehydrogenase belongs to a family of enzymes that exhibits diversity with regard to amino acid composition, cofactor specificity, metal ion requirement and oligomeric state. NADP-linked ICDs have been purified and studied from a variety of eukaryotes and prokaryotes with detail investigations on their subunit composition and kinetic properties [3-11]. ICD from different organisms has been phylogenetically affiliated to three subfamilies [12]. Majority of the bacterial ICDs fall into subfamily I that includes archaeal and bacterial NADP dependent ICDs.

*M. tb *genome has two isoforms of isocitrate dehyrogenase, Rv3339c (ICD-1) and Rv0066c (ICD-2), both annotated as probable isocitrate dehydrogenase based on homology with other enzymes of the ICD family [13]. The two isoenzymes are share only ~14% identity at amino acid level. Earlier, we have pointed to a very unusual property of this TCA cycle enzyme demonstrating that the two isoforms can elicit B cell response in TB patients and significantly discriminate healthy, BCG-vaccinated controls from different groups of TB-infected population when compared to PPD and control antigen *M.tb *HSP 60 [14]. Although the two isoforms have primarily similar antigenic properties, little is known about their enzymatic properties. We now document differences in their biochemical properties, subunit composition and phylogenetic association. Our study provides experimental evidence to show that the two ORFs are TCA cycle enzymes with identical enzymatic function but different physio-chemical and kinetic properties.

## Results

### Expression, purification and quantification of *M.tb *ICD-1 and ICD-2

**Figure 1:**
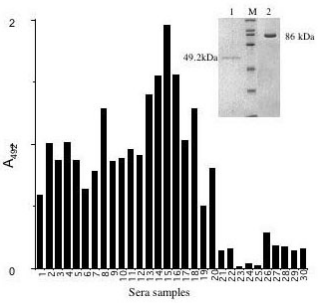
***M. tb *Rv3339c (ICD-1) and Rv0066c (ICD-2) are expressed at the protein level**. Antibody response to *M. tb *ICD-1 and ICD-2 was determined by ELISA. Sample representation of ten *M. tb *patient sera out of 125 showing high immunogenic response when compared with healthy controls. Y axis represents absorbance values at 492λ and X axis represents random patient sera tested for *M. tb *ICD-1 and ICD-2 antigenic response. 1 – 10, antigen ICD-1; 11 – 20, antigen ICD-2; 21 – 25, Healthy controls for ICD-1; 26 – 3, Healthy controls for ICD-2. Inset: Affinity purification of *M. tb *ICD-1 and *M. tb *ICD-2. The different lanes are: lanes 1: *M. tb *ICD-1; lane M: protein molecular size markers (200 kDa, 116 kDa, 97 kDa, 66 kDa, 45 kDa, 31 kDa and 21.5 kDa); lanes 2: *M. tb *ICD-2.

The over-expressed N-terminal His-tagged *M.tb *ICD-1 and *M.tb *ICD-2 were purified on a Nickel affinity column to 95% and 90% homogeneity, respectively (Figure 1, inset). The molecular sizes of the recombinant proteins *M.tb *ICD-1 and *M.tb *ICD-2 were determined by SDS-PAGE analyses and were found to be ~ 49 kDa and ~ 86 kDa respectively. The purification was carried out under native conditions for both the proteins from soluble fractions with an yield of 3.25 mg for *M.tb *ICD-1 and 10.21 mg for *M.tb *ICD-2 per 500 ml start culture [14].

### The ORFs encoding hypothetical protein *M. tb *ICD-1 (Rv3339c) and the probable ICD2 (Rv0066c) are functionally expressed as evident from serological evidences

The *M. tb icd-1 *and *M. tb icd-2*, both are annotated as the probable isocitrate dehydrogenase based on the sequence homology with ICD-family of enzymes. Serological evidences reveal the presence of antibody titers against both the purified proteins ICD-1 and ICD-2 in the infected sera samples [14]. Figure 1 is a sample representation of ten patients each for ICD-1 (sample 1–10) and ICD-2 (sample 11–20) with their respective healthy controls (Figure 1; 21–25, control reactions for ICD-1 and 26–30, control reactions for ICD-2) suggesting that both the ORFs encoding ICDs are expressed at the protein level as evident from antibodies in TB infected patient sera.

### Biochemical characterization reveals differences between ICD-1 and ICD-2 in terms of pH and heat stability

In order to determine the kinetic parameters, the stabilizing components in the reaction like pH, temperature, salt requirement, metal ion components and coenzyme specificity were tested for each enzyme for optimal activity.

**Figure 2:**
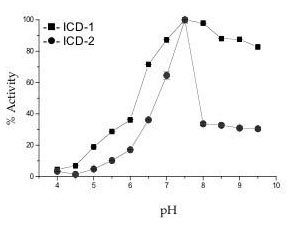
***M. tb *ICD-1 was active across a wider range of pH than *M. tb *ICD-2**. Activity of *M. tb *ICD-1 and *M. tb *ICD-2 was tested as a function of pH (4 to 10). The buffers used for the experiment were: 30 mM Na-acetate buffer (pH 4.0 to pH 5.5), 20 mM phosphate buffer (pH 5.7 to pH 7), 30 mM imidazole buffer (pH 6 to pH 7) and 20 mM Tris buffer (pH 7.5 to pH 10).

Figure 2 shows relative percentage of activity retained by the enzymes with respect to different pH. While the optimum pH for both isoenzyme is 7.5, *M. tb *ICD-1could tolerate a wider pH range retaining ~30% activity in acidic pH 5.5 to ~ 90% activity in alkaline pH 9.5 (Figure 2). In contrast, *M. tb *ICD-2 could retain only 10.22% of activity at a pH of 5.5, which gradually decreased to only 3.4% at pH 4, and less than 40% at 9.5 (Figure 2). These results clearly suggest that *M. tb *ICD-1 is active across a wider range of pH than *M. tb *ICD-2.

**Figure 3:**
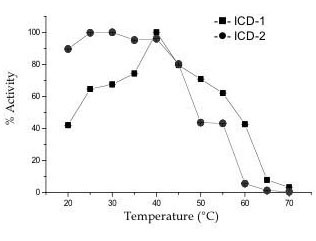
**ICD-1 and ICD-2 exhibit differential activity as a function of temperature**. The enzyme activity was assayed at different temperatures (20°C to 70°C).

Optimum temperature and thermostability of *M. tb *ICD-1 and *M.tb *ICD-2 were studied by varying the temperature of the reaction from 20°C to 70°C with incubation time of 30 minutes for each reaction. The optimum temperature for the activity of *M. tb *ICD-1 is ~40°C (Figure 3) while *M. tb *ICD-2 shows almost similar activity across 20°C to 40°C. Thermal stability of the two enzymes, however, varied. *M. tb *ICD-1 retained ~40% activity till 60°C, where *M. tb *ICD-2 was only ~5% active at that temperature (Figure 3). Thermal inactivation remained irreversible after incubation at 65°C for half an hour for *M. tb *ICD-1. *M. tb *ICD-2 could be renatured upon slow cooling till 55°C where partial activity was restored. Complete denaturation of the protein was observed at about 60°C (Figure 3). Similarity in thermal behaviour of the two enzymes is apparent, however fine differences could be registered.

### The decrease in the activity at higher salt concentration indicates involvement of ionic interactions during catalysis of *M. tb *ICD-1 and ICD-2

**Figure 4:**
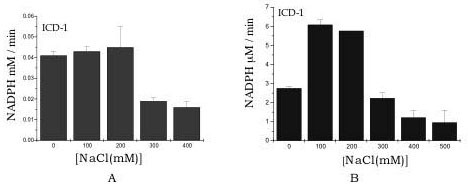
**Activity of *M. tb *ICDs decrease at higher salt concentration**. Activity of the enzymes ICD-1 (a) and ICD-2 (b) was measured by reduction of NADP at different concentrations of NaCl. The enzymes were active in presence of 200 mM NaCl above which the activity rapidly decreases.

We determined the effect of NaCl on the stability as well as activity of the enzymes. The absence of NaCl in the reaction buffer did not affect the activity of *M. tb *ICD-1 significantly but *M. tb *ICD-2 showed higher activity in presence of upto 200 mM of NaCl as compared when no NaCl was added in the reaction. Presence of salt in the reaction buffer probably maintains the integrity of the enzymes. Higher concentrations of salt (above 200 mM) proved to be detrimental for the activities of both the enzymes (Figure 4a and 4b). The purified recombinant proteins, ICD-1 and ICD-2 degraded less at room temperature when dialysed against atleast 100 mM NaCl. The decrease in the activity at higher salt concentration indicates involvement of ionic interactions during catalysis. The reversibility of the inactivation of the enzyme activities after removal of excess salt has not been tested as the enzymes had a tendency to precipitate upon long exposure to room temperature or even 4°C during dialysis.

### *M. tb *ICD-1 and *M. tb *ICD-2 are NADP-dependent and have differential metal cofactors requirement

**Figure 5:**
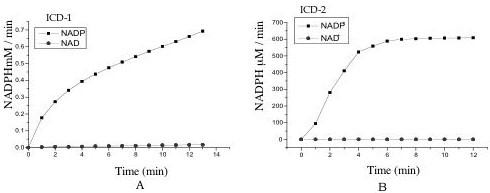
**The *M. tb *ICDs are NADP^+ ^dependent**. Coenzyme specificity of *M. tb *ICD-1(a) and *M. tb *ICD-2 (b) was assayed in presence of NADP^+ ^and NAD^+ ^as coenzymes. The activity curves clearly indicate that *M. tb *ICD-1 as well as *M. tb *ICD-2 are NADP^+ ^dependent enzymes.

The coenzyme specificity of *M. tb *ICD-1 and *M. tb *ICD-2 was confirmed by checking the activity with both NADP^+ ^and NAD^+ ^(Figure 5a and 5b). The activity curves indicate that *M. tb *ICDs are NADP^+ ^– dependent members of the isocitrate dehydrogenase family and shows no activity whatsoever in presence of NAD^+^.

**Figure 6:**
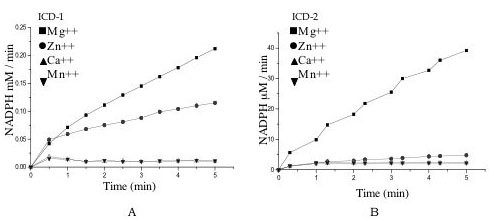
**Comparative rate curves for the enzyme activities in presence of metal ions**. a) Rate curves of *M. tb *ICD-1 and b) Rate curves of *M. tb *ICD-2 in presence of Mg^++^, Zn^++^, Ca^++ ^and Mn^++ ^as cofactors. The details of the reactions are described in the method section.

The two enzymes were tested for metal ion requirement with respect to four divalent metal ions, namely, Mg^++^, Zn^++ ^and Mn^++ ^and Ca^++^. It was apparent that *M. tb *ICD-1 accepts both Mg^++^and Zn^++ ^as divalent metal ion cofactor but shows no activity in presence of either Mn^++ ^or Ca^++ ^(Figure 6a). This is unlike *M. tb *ICD-2, which accepts only Mg^++ ^as metal ion and shows no activity with either Zn^++^, Ca^++ ^or Mn^++ ^(Figure 6b). The saturation kinetics indicates a complete saturation at 10 mM of metal ion for both Mg^++ ^and Zn^++ ^(data not shown).

### Kinetic parameters and feedback inhibition of *M. tb *ICDs

The basic enzyme kinetics parameters Km, Vmax and Kcat for DL-isocitrate and NADP+ were determined for both ICD-1 and ICD-2 (Table 1). Km (isocitrate) in presence of either Mg^++ ^or Zn^++ ^for *M. tb *ICD-1 was calculated to be 10 μM ± 5 and 22 μM ± 7, respectively. For *M. tb *ICD-2, the value is 20 μM ± 1 in presence of Mg^++ ^suggesting that *M. tb *ICD-1 has higher affinity for Mg-substrate complex than

**Table 1:** Kinetic parameters for *M. tb *ICD-1 and *M. tb *ICD-2 with respect to Mg^++ ^and Zn^++^

**Kinetic parameters**	**With MgCl_2_**	**With ZnCl_2_**
***M. tb *ICD-1**		
Km(isocitrate)	10 μM ± 5	22 μM ± 7
Vmax(isocitrate)	380 μM NADPH/min	190 μM NADPH/min
Kcat (isocitrate)	3.8 μM NADPH/min/pM enzyme	1.9 μM NADPH/min/pM enzyme
Km(NADP^+^)	125 μM ± 5	-
Vmax(NADP^+^)	400 μM NADPH/min	-
Kcat (NADP^+^)	4 μM NADPH/min/pM enzyme	-
***M. tb *ICD-2**		
Km(isocitrate)	20 μM ± 1	No activity
Vmax(isocitrate)	371.3 μM NADPH/min	
Kcat (isocitrate)	37.13 μM NADPH/min/pM enzyme	
Km(NADP^+^)	19.6 μM ± 6	
Vmax(NADP^+^)	374 μM NADPH/min	
Kcat (NADP^+^)	37.4 μM NADPH/min/pM enzyme	
Ki(NADPH)	0.46 × 10^-5 ^M.	

**Figure 7:**
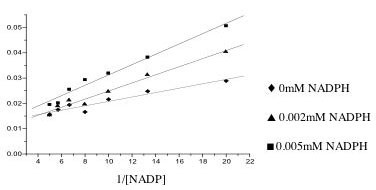
**Lineweaver Burk plot for competitive inhibition of *M. tb *ICD-2**.

Zn-substrate and also more readily binds to metal-substrate complex than *M.tb *ICD-2. The observation that these enzymes showed no activity upon pre-incubation with isocitrate and NADP^+ ^in absence of metal ions suggests that they are unable to utilize free isocitrate as a substrate. Km (NADP) values in presence of Mg^++ ^for *M.tb *ICD-1 and *M. tb *ICD-2 are 125 μM ± 5 and 19.6 μM ± 6, respectively. Competitive inhibition was observed with NADPH versus NADP^+^for *M. tb *ICD-2 (Figure 7). The mean inhibitor constant, Ki, was calculated to be 0.46 × 10^-5 ^M. Competitive inhibition of *M.tb *ICD-2 by NADPH indicates the regulation of this enzyme by feedback mechanism (Figure 7). Since *M. tb *ICD-1 showed very poor affinity for NADP^+ ^(125 μM ± 5), similar feedback inhibition was not observed.

### Oligomeric assemblies of *M. tb *ICDs

**Figure 8:**
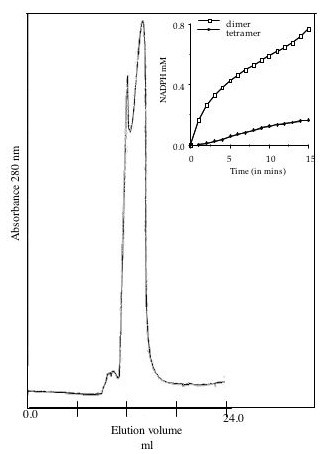
***M. tb *ICD-1 is a homodimer as evident from gel filtration analysis**. The elution profiles of recombinant *M. tb *ICD-1 on a Superdex-200 HR 10/30 column showed two peaks corresponding to tetrameric (200 KDa) and dimeric (100 KDa) state of the protein. Inset: Comparative activity of the collected fractions.

Size exclusion chromatography was performed to check the oligomeric assembly of the recombinant *M.tb *ICD-1 and *M.tb *ICD-2. The chromatogram for *M.tb *ICD-1 showed two distinct peaks indicating presence of two oligomeric species in the solution (Figure 8). The first peak corresponds to a mass of ~200 kDa (tetrameric) while the major peak showed migration of the molecule as a mass of ~100 kDa (dimeric). The results show *M.tb *ICD-1 to exist in either minor tetrameric or major dimeric state. In order to evaluate the functional significance of the two oligomeric states in catalysis of *M.tb *ICD-1, each fraction was collected separately and checked for the activity. While the dimeric fraction showed complete activity, the tetrameric fraction displayed a rather insignificant activity (Figure 8, inset). A few dimeric species that might have originated due to distintegration of the tetrameric form may account for the insignificant activity of the collected tetrameric fraction. The monomeric form was previously checked for the activity and was found to be inactive (data not shown). Thus, the oligomeric functional form of the *M.tb *ICD-1 is a dimer.

**Figure 9:**
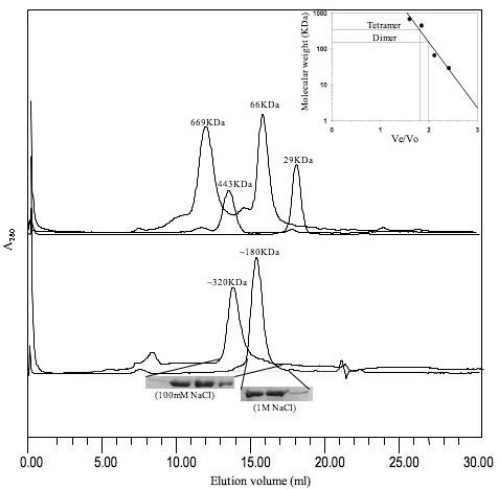
**Originally annotated as a monomer, *M. tb *ICD-2 exists as a homodimer**. The elution profiles of recombinant M.tb ICD-2 on high performance column SuperoseTM 6 10/300 column. The lower panel of the chromatogram shows a peak corresponding to a tetramer (~320 KDa) under low salt condition, which was dissociated into a dimer (~180 KDa) at a high salt concentration of 1 M. The collected elution fractions run on 7% SDS-PAGE are shown under respective peaks. The upper panel represent chromatographic peaks corresponding to the protein markers; Thyroglobulin (669 KDa), Apoferritin (443 KDa), BSA (66 KDa) and Carbonic anhydrase (29 KDa). Inset: The calibration curve plotted as Ve/Vo versus log of molecular mass for calculating molecular weights of the oligomeric assembly.

The gel filtration analysis were performed with high performance column Superose™ 6 10/300 GL for higher resolution using an automated chromatographic workstation (BioRad BioLogic Duoflow™) to confirm the oligomeric state of *M. tb *ICD-2. The experiments were performed with the concentrated protein (~400 μg) under both low (100 mM NaCl) and high (1 M NaCl) salt conditions. The chromatogram showed a peak corresponding to a tetramer (~320 KDa) under low salt condition, which was dissociated into a dimer (~180 KDa) at a high salt concentration of 1 M, but not into a monomer (Figure 9, lower panel). The broadening of the ICD-2 peak under low salt concentration at higher elution volume points to the existence of lower oligomeric species. Sharpness of the peak under 1 M salt corresponding to a dimeric size provides strong evidence that the most stable oligomeric state is a dimer.

**Figure 10:**
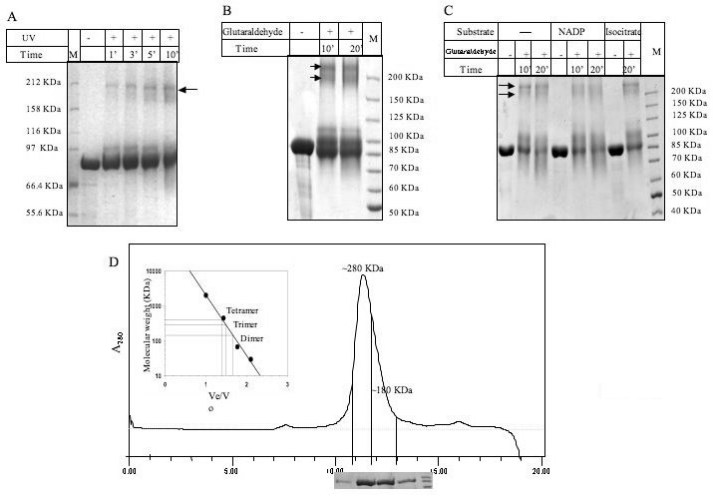
**Crosslinking profiles of M. tb ICD2**. A. UV crosslinking profile, B. Chemical crosslinking using glutaraldehyde, C. Glutaraldehyde crosslinking with substrate (Isocitrate) and coenzyme (NADP). UV crosslinking profile clearly shows a band corresponding to a homodimeric species. Chemical crosslinking with glutaraldehyde in presence and absence of NADP and Isocitrate was more efficient and showed bands corresponding to both dimer and trimer forms of M.tb ICD 2. D. The gel filtration assay under an intermediate (500 mM) salt concentration revealed a peak representing a mixture of trimeric and dimeric species. The collected elution fractions were run on 7% SDS-PAGE. D (Inset): The calibration curve plotted as Ve/Vo versus log of molecular mass for calculating molecular weights of the oligomeric assembly.

These conclusions were further supported by UV (figure 10a) and chemical crosslinking data (figure 10b). It was observed that UV crosslinking gave a distinct dimeric species. However, chemical crosslinking with glutaraldehyde was more efficient and it showed bands corresponding to both dimer and trimer forms of *M. tb *ICD2.

We also performed chemical crosslinking in presence and absence of coenzyme (NADP) and substrate (Isocitrate). A band corresponding to a trimer was consistently present with the dimeric band (figure 10c). Since the gel filtration data under low salt condition showed a tendency of forming lower oligomeric states, we further explored if under any salt condition a trimeric species that was observed in crosslinking could be obtained. Therefore the gel filtration assay was repeated under an intermediate (500 mM) salt concentration. Figure 10d shows a peak representing a mixture of trimeric and dimeric species under this buffer condition. Therefore, we conclude that *M. tb *ICD-2 is not a monomeric protein under any conditions tested in this study. Eventhough it can exist in higher oligomerc forms, dimer represented the most stable form that could not be further dissociated into monomers in our buffer condition. The above result equivocally demonstrate that *M. tb *ICD-2 is not a monomer and exists in higher oligomeric state in contrary to what has been reported in the annotation of H37Rv genome.

### *M. tb *ICD-1 and *M. tb *ICD-2 have different phylogenetic affiliations

**Figure 11:**
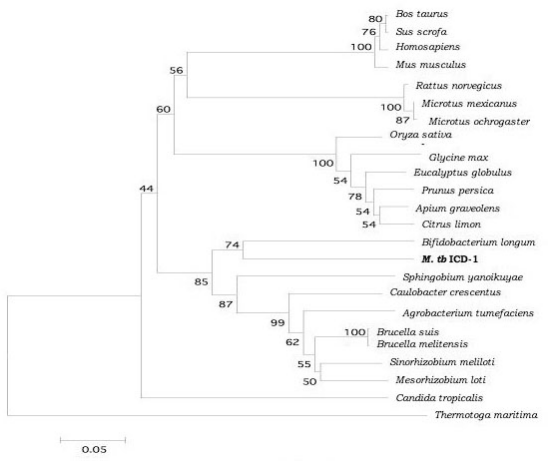
***M. tb *ICD 1 is closer to eukaryotic NADP^+ ^ICDs**. A 50% consensus bootstrap neighbour joining tree of *M. tb *ICD-1 with *Thermotoga maritima *as outgroup. The numerical values represent the confidence assessed by bootstrap analysis. The tree depicts a closer lineage of *M. tb *ICD-1 with eukaryotic NADP dependent isocitrate dehydrogenases. The amino acid sequence alingnment of ICDs from these organisms showed more than 65% identity with all the major catalytically important residues conserved (refer text).

**Figure 12:**
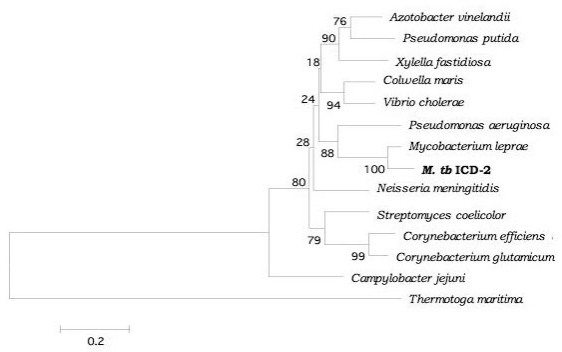
***M. tb *ICD-2 is closer to prokaryotic NADP^+ ^ICDs**. Rooted neighbour joining phylogenetic tree of *M. tb *ICD-2 with *Thermotoga maritima *as outgroup. The numerical values represent the confidence assessed by bootstrap analysis. ICD-2 clusters with NADP^+ ^dependent ICDs of gram-ve bacteria. *M. tb *ICD-2 has closest homology with *Mycobacterium leprae *showing 85.4% similarity at protein level. Other close neighbours are human pathogens like *Pseudomonas aeruginosa *(65%), *Vibrio cholerae *(60%), *Neisseria menengitidis *(59%) etc.

The ICD-1 and ICD-2 were aligned at the protein sequence level, with the corresponding sequences from a range of organism from both prokaryotes and eukaryotes, after a BLAST search (data not shown, but available upon request). Based on the sequence alignment using CLUSTAL, phylogenetic analyses were carried out. Neighbour Joining rooted trees with *Thermotoga maritima *isocitrate dehydrogenase as outgroup were constructed for both ICD-1 and ICD-2. The confidence was assessed by bootstrap analysis as described in Materials and Methods. The results reveal a closer relationship of the functionally conserved residues of *M. tb *ICD-1 with eukaryotic NADP^+ ^dependent isocitrate dehydrogenases (Figure 11). The closest prokaryotic neighbour of *M. tb *ICD-1 was *Bifidobacterium longum*. The NADP-dependent isocitrate dehydrogenases of the following prokaryotes were found to cluster with *M. tb *ICD-1: *Spingobium yanoikuyae, Caulobacter crescentes, Agrobacterium tumefacians, Brucella sp., Sinnorhizobium meliloti *etc (Figure 11). *M. tb *ICD-1 showed no similarity with any of the mycobacterial ICDs in the protein database, including the second isoform of *Mycobacterium tuberculosis, M. tb *ICD-2. The phylogenetic tree of *M. tb *ICD-2 however, is a total contrast of *M. tb *ICD-1, where it clusters with other NADP+ dependent ICDs of gram-ve bacteria (Figure 12).

## Discussion

Our results demonstrate for the first time experimentally that the two *M. tb *ORFs Rv3339c and Rv0066c code for functional TCA cycle enzyme that can catalyze the conversion of D-isocitrate to α-ketoglutarate and CO_2 _in presence of NADP as cofactor and differ in their biochemical properties. Interestingly, an altogether different functionality of these two isoforms in immune recognition was evident from our earlier work [14]. We have biochemically characterized the two isoforms of *M. tb *ICDs and established the differences between them.

Isocitrate dehydrogenase is an important regulatory enzyme of TCA cycle that has been intensively studied in both prokaryotes and eukaryotes (*3–11*). It lies at the branchpoint between glyoxylate shunt and citric acid cycle in prokaryotes where the switchover from TCA cycle to glyoxylate shunt depends upon the alteration in the biochemical parameters of isocitrate dehydrogenase. Isocitrate lyase of glyoxylate shunt pathway has much lower affinity for the isocitrate and cannot compete with isocitrate dehydrogenase for the substrate under normal conditions. It has been reported that phosphorylation of ICD controls the flux of isocitrate between the Krebs cycle and the glyoxylate pathway [15,16]. In *E.coli*, where glyoxylate bypass and citric acid cycle operate concurrently, the activity of a single, functional isocitrate dehydrogenase is closely monitored [17,18]. In *M. tb*, however, glyoxylate bypass is observed inside macrophages where C_2 _substrate is the main carbon source [19]. The occurrence of two isoforms of ICD in *M. tb *genome with the possibility of each having characteristic biochemical properties is interesting under such circumstances.

*M. tb *ICD-1 and ICD-2 follow a first order reaction and exhibit typical saturation kinetics. Km (isocitrate) value for *M. tb *ICD-1 clearly indicates a high affinity of this enzyme for isocitric acid as compared to *M. tb *ICD-2. Km (isocitrate) of some of the known NADP^+ ^dependent ICD has been presented in Table 2 for reference and comparison [3-11]. Several other probable substrates for *M. tb *ICD-1 with close structural similarity were tested. Two such substrates were aspartate and glutamate. Both the compounds have close similarity with isocitric acid. However, the poor activity of the enzyme with these substrates confirmed that the enzyme is specific to only isocitrate as a substrate (data not shown).

**Table 2:** Comparison of NADP^+^-dependent isocitrate dehydrogenases from different organisms.

Organism	Km(isocitrate)	Km(NADP^+^)
*Blastocladiella emersonii*	20 μM	10 μM
*Chlorobium limicola*	45 μM ± 13	27 μM ± 10
*Bacillus substilis*	5.9 μM ± 0.9	14.5 μM ± 2.2
*Escherichia coli*	4.9 μM ± 0.2	19.6 μM ± 3.6
*Synechocystis sp PCc *6803	59 μM	12 μM
*Pyrococcus furiosus*	-	4400 μM
*Aeropyrum pernix*	-	30 μM
*Thermotoga moritima*	-	55.2 μM
*Aeropyrum fulgidus*	-	30 μM
*Mycobacterium phlei *(ATCC-354)	74 μM	53 μM
***M. tb *ICD-1**	10 μM ± 5	125 μM ± 5
***M. tb *ICD-2**	20 μM ± 1	19.6 μM ± 6
Beef liver NADP^+^-IDH	1.7 μM	7.3 μM
Rat liver (cytosolic)	9.7 μM ± 2.9	11.5 μM ± 0.2
Porcine heart NADP^+ ^IDH	-	5 μM ± 0.19

The fact that *M. tb *ICD-1 could tolerate a broad range of both pH and temperature (Figure 2, 3) than *M. tb *ICD-2 indicates its robustness. The difference in the pH tolerance helps to postulate the possibility of differential expression of the two isoforms with ICD-1 being expressed during stationary phase when the intracellular pH is expected to vary over a wider range than log phase.

Km (NADP+) of *M. tb *ICD-1 showed poor affinity for NADP+ as compared to *M. tb *ICD-2 and other known NADP-dependent isocitrate dehydrogenases (Table 2). The poor affinity of *M. tb *ICD-1 to NADP^+ ^warranted an investigation on whether dual co-enzyme specificity occurs in *M. tb *ICD-1 as reported in some archaeal bacteria [12]. We, therefore, compared the enzymatic activity of both the enzymes in presence of NADP^+ ^and NAD^+ ^(Figure 5a and 5b). It can be clearly seen that *M. tb *ICD-1 as well as *M. tb *ICD-2 accepts NADP^+ ^and not NAD^+ ^as a proton acceptor.

The homodimeric state of *M. tb *ICD-1 is the functionally active species, even though residual activity was noticed in tetrameric fraction which could be a reflection of the presence of a few dimeric species as a consequence of disintegration of the tetrameric forms. The chromatogram peak for *M. tb *ICD-2 corresponding to a tetramer (~320 KDa) under low salt condition, which was dissociated into a dimer (~180 KDa) at a high salt concentration of 1 M, but not into a monomer (Figure 9) provided a strong evidence that the most stable form of *M. tb *ICD-2 is a dimer. An intermediary trimeric form was observed in chemical crosslinking assays (Figure 10b and 10c), both in presence and absence of coenzyme NADP and substrate isocitric acid. The data were consistent with the gel filtration profile under an intermediate (500 mM) salt concentration (Figure 10d) where it showed a peak representing a mixture of trimeric and dimeric species. We therefore could conclude that *M. tb *ICD-2 is not a monomeric protein. Our result indicates *M. tb *ICD-2 exists in different higher oligomeric states which may follow the following equilibria: [tetramer] ⇔ [trimer] ⇔ [dimer]. However, the physiological relevance of the different oligomers could not be concluded from our experiments.

Earlier attempt to trace the evolution of ICDs to understand the adaptive role of isocitrate dehydrogenase in intracellular persistence of this pathogen by Steen et al [12] does not place *M. tb *ICD-2 phylogenetically. Proximity of the two *M. tb *ICDs with other isocitrate dehydrogenases was determined. Our results on phylogenetic analysis of *M. tb *ICD-1 revealed a closer relationship with eukaryotic NADP^+ ^dependent ICDs (Figure 11) with more than 65% identity with that of *Glycine max, Sus scrofa, Bos *and *Homo sapiens*. *M. tb *ICD-1, indeed, is correctly placed in subfamily II that includes eukaryotic NADP dependent ICDs and a single bacterial ICD (*Sphingomonas yanoikuyae*) [12]. With NADP+ dependent isocitrate dehydrogenase of *Sphingomonas yanoikuyae*, *M. tb *ICD-1 has more than 65% similarity at primary structure level. Phylogenetic analysis of *M. tb *ICD-2 showed that the classical nomenclature applies to ICD-2 and it can be placed in subfamily I, the closest being *M. leprae *(Figure 12). The closest bacterial relative of *M. tb *ICD-1 as inferred by our study is NADP dependent isocitrate dehydrogenase of *Bifidobacterium longum *(Figure 11). *Bifidobacterium *sp. are gram positive, anaerobic, natural components of human intestinal microbiota [20]. This might be argued as a case of horizontal transfer or lateral transfer of gene amongst unrelated organisms across the boundaries of phylogenetic domains. Horizontal transfer of genes is a common occurrence in nature and accounts for almost 10–50% of genes in bacteria [21,22].

## Conclusion

Several ORFs have been characterized since deciphering of *M. tb *genome [23-25]. Our data represent conclusive proof that the two ORFs, Rv3339c and Rv0066c, are functional TCA cycle enzyme and represent the first attempt to characterize these important members of the TCA cycle of *Mycobacterium tuberculosis*. Our studies conclusively reveal that both ICD-1 and ICD-2 are NADP^+ ^dependent members of ICD family with the former having closer homology with eukaryotic ICDs and latter with prokaryotes. ICD-1 is a homodimer, while ICD-2 annotated as a monomer, exists in higher oligomeric forms, the dimer being the most stable. The two isoforms differ in their affinity for coenzyme NADP as represented by their Km(NADP) values (Table 1) and also with respect to pH tolerance and thermostability.*M. tb *ICD-2 is a more efficient enzyme as inferred by comparing Vmax(NADP)/Km(NADP) ratios for the two enzymes but *M. tb *ICD-1 is more robust in terms of pH tolerance and thermostability. The possibilities of differential expression of these two isoforms during different stages and conditions of growth cannot be ruled out even though the two isoforms have identical enzymatic function.

## Methods

### Cloning and purification of *M. tb *ICDs

The 1.230 kb (ICD-1) and 2.238 kb (ICD-2) long ORF was amplified from H37Rv genomic DNA and overexpressed in the pRSET-A/*E.coli *BL-21 (DE3) expression system as described earlier [14]. The overexpressed his-tagged recombinant protein was purified by Ni^2+^-nitrilotriacetate affinity chromatography.

### Enzyme linked immunosorbent assays (ELISA)

ELISAs were performed with purified recombinant proteins, as described earlier [14], to check the B cell immune response in TB patient sera as evidence to the *in vivo *expression of the proteins.

### Dehydrogenase kinetics/biochemical assays

Dehydrogenase activity was measured spectrophotometrically by monitoring the time dependent reduction of NADP+ to NADPH at 25°C in Unicam UV/Vis spectrometer at 340 nm, the absorbance maximum of NADPH. The standard assay solution contained 20 mM triethanolamine chloride buffer pH 7.5, 2 mM NADP+, 0.03 mM DL-isocitrate, 10 mM MgCl2/10 mM ZnCl2, 100 mM NaCl and 10 -100 pM of the enzyme in a final volume of 400 μL. Environmental parameters for the enzymes were measured by altering the pH of the buffer (range 4 – 10), temperature (20 – 65°C), concentration of substrate (0.01 mM – 0.18 mM), cofactor (0.1 – 2 mM), metal ion (Mg++, Zn++, Mn++; 1 – 12.5 mM) and salt (100 mM – 500 mM) requirement (as indicated in the respective figure legends. The pH dependence of the enzyme was measured using the following buffers: 30 mM Na-acetate buffer (pH 4.0 to pH 5.5), 20 mM phosphate buffer (pH 5.7 to pH 7), 30 mM imidazole buffer (pH 6 to pH 7) and 20 mM Tris buffer (pH 7.5 to pH 10). The cofactor specificity was checked with both NADP+ and NAD+. The heat denaturation was studied in 20 mm triethanolamine chloride buffer pH 7.5 in presence of 1% bovine serum albumin. Enzyme aliquotes were placed in tubes and incubated for 30 minutes in a water bath set at the required temperature (20°C – 65°C). After heating, aliquotes were immediately placed on ice and then assayed for remaining enzyme activity.

The kinetic analysis was carried out at 25°C and pH 7.5 in presence of either Mg^++ ^or Zn^++^. Km was determined by altering the concentration of either the substrate or the coenzyme. The substrate concentration gradient varied from 0.01 mM to 0.75 mM, while NADP+ concentration was taken from 0.1 mM to 2 mM. The values were plotted as V vs S for calculating Km and Vmax for this first order reaction. The results were counter checked by double inverse Lineweaver-Burk plot. Competitive inhibition was observed with reduced NADP (NADPH) versus NADP to estimate inhibitor constant, Ki. Standard Km analysis was performed followed by repeating the assay with NADPH. Two concentrations of the inhibitor were tested, 0.002 mM and 0.005 mM. The uninhibited run provided the value of Km for the reaction and the inhibited run provided the apparent Km (Kmapp) for the reaction. Ki for the competitive inhibition was calculated by the formula (Km) (I)/Kmapp – Km.

### Size exclusion chromatography

Size exclusion chromatography was performed at room temperature using FPLC equipped with Superdex-200 HR 10/30 column (Amersham Pharmacia Biotech) and SuperoseTM 6 10/300 GL (BioRad BioLogic Duo-flow™). Calibration of the columns were performed using protein molecular-mass standards for gel-filtration (Sigma, USA) as described elsewhere [26]. The void volume (V_o_) was determined by running Blue Dextran on the column. The calibration curve was plotted as Ve/Vo versus log of molecular mass. A 2.4 mg/ml (for ICD-1) and 1.06 mg/ml (for ICD-2) concentrations of recombinant proteins were used for all gel filtration experiments. The columns were equilibrated with three bed volumes of the elution buffer prior to each run. Protein elution was monitored at A_280_.

### UV and chemical crosslinking

UV crosslinking assays were performed to check the oligomeric assembly of *M. tb *ICD2. 5 μg of protein per reaction, in TrisCl buffer pH 8, was taken and exposed to UV in a UVP CL-1000 Ultraviolet crosslinker for 1 to 10 minutes at the rate of 1600 Joules/minute and fractioned later on 10% SDS-PAGE along with similar amount of untreated *M. tb *ICD2 as control. For chemical crosslinking, the protein was equilibrated in 20 mM phosphate buffer pH7.8. 10 mM Glutaraldehyde was used for all the chemical crosslinking reactions. The protein samples were incubated at 37°C, with or without glutaraldehyde. The reaction was stopped at different time points (10' or 20') using SDS loading dye containing 400 mM glycine and subsequently fractionated on 7% SDS-PAGE. 1 mM of either NADP or Isocitrate was used wherever required.

### Sequence alignment and phylogenetic analysis

The amino acid sequence of *M.tb *ICD-1 and *M.tb *ICD-2 were compared against the NCBI protein database [27]. The sequences with the BLAST score upto e-153 or 65% identity were selected for construction of the phylogenetic tree. The sequences were aligned using CLUSTAL program. Manual alignment was done by Jalview [28] wherever required. The sequence alignment is available on request from the authors. Rooted phylogenetic tree were constructed using the software MEGA3 [29] using the amino acid sequence of *Thermotoga maritima *isocitrate dehydrogenase as outgroup. The confidence was assessed by bootstrap analysis (thousand replicates using default parameters).

## List of abbreviations

ICD, isocitrate dehydrogenas; *M. tb, Mycobacterium tuberculosis*; TCA, tricarboxylic acid; NADP, nicotinamide adenine dinucleotide phosphate; BSA, Bovine Serum Albumin; NAD, nicotinamide adenine dinucleotide; NADPH, reduced nicotinamide adenine dinucleotide phosphate; bp, base pair(s); kb, kilobase pair(s); kDa, kilo Daltons, pM, pico molar; μM, micro molar.

## Authors' contributions

SB has contributed in conception and designing of all experiments, biochemical assays and *in-silico *analysis, interpretation of data and writing the draft manuscript. AN carried out the biochemical assays and, participated in the sequence alignment and phylogenetic analysis. RP did the cloning and purification of proteins. VMK was involved in overall designing of this study, critical analysis of the data and also provided the biological material and quality control. SEH have been involved in complete supervision and guiding the work, providing the intellectual inputs, revising the draft version and preparing the final version of the manuscript.
